# *QuickStats:* Number of Deaths from Hornet, Wasp, and Bee Stings* Among Males and Females — National Vital Statistics System, United States, 2011–2021

**DOI:** 10.15585/mmwr.mm7227a6

**Published:** 2023-07-07

**Figure:**
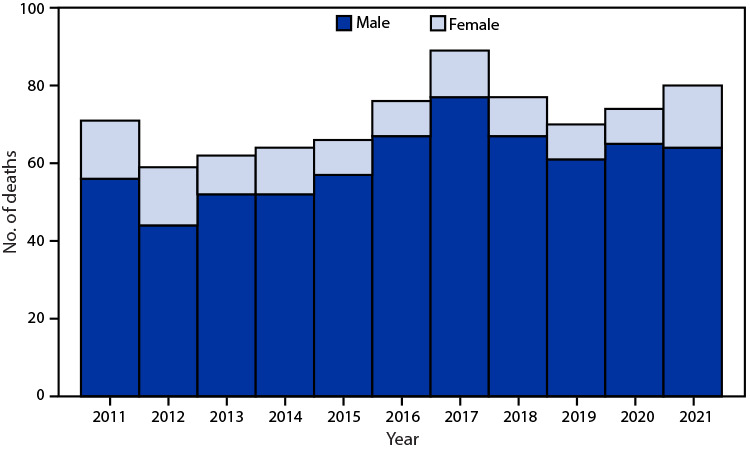
During 2011–2021, a total of 788 deaths from hornet, wasp, and bee stings occurred (an average of 72 deaths per year). The annual number of deaths ranged from 59 (2012) to 89 (2017). Overall, 84% of deaths occurred among males.

**Source:** National Center for Health Statistics, National Vital Statistics System, Mortality Data, 2011–2021. https://wonder.cdc.gov/Deaths-by-Underlying-Cause.html

